# New perspectives on repetitive behaviour

**DOI:** 10.1007/s00426-025-02092-6

**Published:** 2025-03-06

**Authors:** Faith Jeremiah, Russell Butson, Adekunle Oke

**Affiliations:** 1 Faculty of Agribusiness and Commerce, Lincoln University, PO Box 85084, Canterbury, 7647 New Zealand; 2 Higher Education Development Centre, University of Otago, Dunedin, New Zealand; 3 Leeds Business School, Leeds Beckett University, Leeds, LS1 3HL UK

## Abstract

Human existence is shaped by interconnected patterns and repetitions that unfold in rhythmic cycles, from biological functions to socially constructed behaviors. While innate (physiological) and natural (environmental) cycles remain fixed, self-constructed cycles, such as routines and habits, are more dynamic, shaped by human agency. These repetitive behaviors often enhance efficiency, goal achievement, well-being, and stress reduction. However, over-reliance on them can lead to rigidity, inhibiting innovation, serendipity, and adaptability. In an era of rapid technological change, the highly routinized lifestyle of the industrial age may no longer be optimal. Drawing insights from a systems-thinking perspective, we reconceptualize routines and habits as dynamic constructs that offer both stability and adaptability in shaping human behavior. This paper contributes to the academic discourse on temporal structures and innovation by critically examining how routines function as both enablers and constraints in a rapidly evolving world, offering practical insights into fostering greater flexibility in behavioral and adaptive patterns.

## Introduction

The chains of habit are too weak to be felt until they are too strong to be broken.

(Johnson, 1847, p. 117).

Have you ever tried to break the habit of waking up and immediately checking your phone? This challenge arises because habits, triggered by context or environmental cues, psychological patterns, and physiological responses (e.g., affective rewards), are deeply ingrained in human behaviour.

Habits and habit formation research has been traditionally confined to human psychology; however, the trend has gradually diffused into many disciplines, including ecology and business management (Gallimore & Lopez, 2002). The dominant school of thought suggests that habits, when formed through daily routines, shape human behaviour and how they interact with their environment. Evidence (Cohn & Lynch, 2017; Ehn & Löfgren, 2020) further indicates that when this pattern is disrupted or life dramatically shifts, we quickly and impulsively seek to establish new routines and habits in a new environment. The process of establishing new routines and its outcomes indicate that almost half of our activity can be automatic (Wood et al., 2002). The assumption that we can entirely control and shift our habitual and routinised behaviour (see Cohn & Lynch, 2017, for example) raises significant complexities when we unpack the fixed and innate patterned cycles and rhythms interlinked with human psychology and behavioral structures.

Indeed, a plethora of research (Kruglanski & Szumowska, 2020; Pedersen & Muhr, 2021) has sought to unpack such complexities, leaving an assemblage of agreements and debates. Many of these agreements and debates appear more culturally appropriate to the industrial age. They are considered traditional or cultural due to the shared beliefs of communities whereby these activities shape our behaviour and are habitual parts of daily routines (Gallimore & Lopez, 2002).

Thus, differentiating between routines and habits has also been part of an ongoing dialogue across disciplines. Foundationally, routines are accepted as time-bound and deliberate, structured around explicit goals and allowing for flexibility in response to changing circumstances (Bernacer & Murillo, 2014). Habits, on the other hand, are context-bound (environmental), automatic responses requiring minimal cognitive engagement (Wood & Rünger, 2016). These differences have been attributed to variations in goal-directed behaviour, reflexivity, and cognitive engagement. To illustrate these distinctions, consider the practice of driving to work. The act of commuting at a specific time each day (navigating predictable traffic patterns) is often framed as a routine, tied to time and deliberate planning. However, the reflexive behaviour of fastening a seatbelt immediately upon entering a vehicle exemplifies a habit cued by environmental triggers rather than time. This dynamic interplay underscores how routines and habits can coexist and influence broader behavioural patterns.

Yet fully appreciating this interplay remains challenging, as some academics continue to support routines and habits as distinct constructs (Kruglanski & Szumowska, 2020). However, associative learning theorists challenge these distinctions, arguing that temporal features of a context are as important as the sensory features (e.g., Urcelay & Miller, 2014), rendering the distinction between context-bound and time-bound behaviours meaningless.

By grounding our argument in the above literature, we set the stage for a nuanced exploration of routines and habits, emphasizing their relevance for innovation and adaptability. We examine the complex relationship between routines and habits by distinguishing three dimensions of patterned activity: innate, natural, and constructed. Innate rhythms, such as circadian cycles, and natural environmental patterns, like seasonal changes or tidal movements, are not behaviors themselves but rather fundamental forces that shape human activity. In contrast, constructed routines and habits emerge through personal, social, and cultural influences, allowing for flexibility and adaptation over time. These human-driven patterns can both constrain and enable behavior, influencing goal achievement, creativity, and adaptability. By unpacking these dimensions, we posit that routines and habits interact within a broader framework of human behavior, offering both stability and opportunities for innovation.

This paper aims to reconceptualise routines and habits by establishing clear distinctions between them, examining their interplay, and discussing their implications for adaptability in the digital age. This conceptual reframing contributes to the discourse on temporal literature and innovative research while offering insights into fostering proactive, adaptive behaviours and mindsets. This paper challenges conventional thinking, seemingly preoccupied with championing rigid, repetitive behaviour. However, in today’s digitally driven modern era, traditional paradigms may no longer suffice; increasingly fluid and digitalised environments now demand greater flexibility in routines and habits.

## Conceptual framework and purpose

This paper positions routines and habits as dynamic constructs, conceptualising routines and habits as a dynamic form of human instinct. Unlike fixed animal instincts, routines and habits are uniquely human in their ability to be constructed, shaped, and adapted. This dynamic quality allows them to serve dual roles: offering stability during disruption while enabling intentional adaptability relevant to fostering proactive, adaptive behaviours and mindsets. In response, this paper seeks to address critical research questions:

- How do routines and habits contribute to proactive behaviours and flexibility in fast-changing environments?
- What practical strategies can transform maladaptive patterns into adaptive ones, fostering growth and innovation?

By reframing routines and habits in this way, this paper transcends a descriptive approach, offering actionable insights into leveraging these patterns for innovation and growth. It does this by recognising that routines stabilise behaviour and reduce cognitive load while they anchor individuals in rigidity. Strategies that consciously disrupting routines with exploratory activities or embedding micro-habits designed for adaptability are pivotal in this regard.

Linking this analysis to broader adaptive behaviours, such as the distinction between fixed and growth mindsets (Dweck, 2006), is helpful in understanding its relevance and impact. Fixed routines may reflect a static approach to problem-solving, whereas growth-oriented habits promote proactive adaptation and resilience. This dynamic interplay not only enriches the theoretical framework but also positions routines and habits as central to fostering innovation and adaptability in rapidly changing contexts.

## Exploring the landscape of routines and habits

The various perspectives, tensions, and debates on routines and habits in the literature highlight the complexity of unpacking patterned and rhythmic cycles. Diverging into the full array of scholarly tensions is not the focus of this paper. Instead, it is designed to highlight some prominent, potentially outdated, schools of thought and demonstrate the importance of a new conceptual understanding consistent with the modern digital age. For example, there is an overarching academic agreement (e.g., Bernacer & Murillo, 2014; Lima et al., 2016; Mendelsohn, 2019; Neuberg & Newsom, 1993) that habits are unconscious and context-dependent and that routines are consciously bound by time. However, there is no clarity around human reflexive capabilities to accurately account for when and why habits trigger conscious thought, making the idea of habits as a form of repetitive behaviours questionable and open for debate.

Here, we argue that this traditional viewpoint overlooks their interconnectivity and fails to discuss other constructed patterns, such as whether rituals are a precursor or interrelated with routines and habits. The literature emphasises the psychological aspects of habits and routines (Wood & Rünger, 2016) while underestimating the impact of the physiological and natural dimensions that influence constructed patterned behaviour. Around these debates are the often-overlooked dynamic contextual states, with the digital dimension now increasingly coinciding with our physical realm.

This point overlaps with another potentially overlooked area—the application of systems thinking as a conceptual framework for understanding how routines and habits interact and function within human behavior. While this paper does not engage in a formal systems-theory analysis, it adopts a metaphorical systems-thinking perspective to illustrate how routines and habits operate as dynamic and interwoven processes rather than isolated behaviors. Systems thinking, in this metaphorical sense, contrasts with linear thinking by embracing a holistic, integrative approach rather than an analytical, dissected one (Monat & Gannon, 2015). It highlights how recurring patterns emerge not in isolation but within interdependent structures shaped by psychological, physiological, and social influences. This perspective prioritizes the study of relationships over individual components and recognizes self-organization and emergence as key factors in behavioral adaptation. This approach will be explored further in the discussion.

## Traditional views on habits and routines

The traditional academic consensus (e.g., de Wit et al., 2009; Ehn & Löfgren., 2020; Galla & Duckworth, 2015; Keller et al., 2021; Quinn et al., 2010; Verplanken & Aarts, 1999) positions habits as an unconscious substrate of human behaviour. These scholars explain that given enough repetition, habits become a motor or cognitive recurrence that can complete itself without conscious supervision (Bernacer & Murillo, 2014 p. 883). In this context, habits generate an impulse to behave or act with limited or no deliberate thought processes (González et al., 2008). While behaviour can be automatic without being planned or deliberate, such behaviour is predicated on how accessible beliefs (personal or cultural) and experiences are in human memory. To illustrate this line of thinking, consider this scenario:

Every day, over the last two years, John runs ten 3km laps at 6 am. Today, after completing his ten laps, like always, John runs across to the side bench and, without looking, grabs his towel from where it normally sits and wipes the sweat off his face. Within seconds, he recoils in horror as he realises he has grabbed another runner’s towel. It was not that John had forgotten the colour of his towel; it was that for the last two years, every day after his run, John would grab his towel from the same bench post without conscious thought or reasoning.

This mini-narrative illustrates popular thinking, suggesting that habits, once formed, become unconscious behaviour (Bernacer & Murillo, 2014; Lima et al., 2016; Mendelsohn, 2019; Neuberg & Newsom, 1993). This notion conflicts with our sense of cognitive independence and free will to adjust our behaviours based on contextual cues. As Mendelsohn (2019) explains, despite our sense of control and intentions in life, a significant amount of our behaviour occurs and is even formed by habit without our awareness. It is fair to say that drawing attention to these unconsciously formed behaviours may be the starting point for change and adjustment.

If we accept that habits are context-dependent (Bernacer & Murillo, 2014; Lima et al., 2016; Mendelsohn, 2019), then we would agree that repetition and associations with cues from the surrounding environment would solidify them. Therefore, once habits are formed, the perception of the cue (in John’s case, the cue of finishing the track) would be sufficient to trigger the response (grabbing the towel) automatically. The illustrative case suggests our dynamic world with a constantly changing environment can alter our cognitive capabilities to retrieve information automatically and apply it accurately when performing routine or mundane actions. However, scholars would agree that habits require less cognitive input because they are performed quickly and automatically and, as a result, tend to be relatively inflexible (Wood & Rünger, 2016).

Contrary to habits, routines are typically framed as conscious and reasoned behaviour or repetitive cycles bound by time (Bernacer & Murillo, 2014; Lima et al., 2016; Mendelsohn, 2019; Wood & Rünger, 2016). Examples of routines include going to the gym at 6 am daily, buying coffee every morning at a particular café, a bi-weekly store visit, or daily watching the 6 pm news. Following this line of thought, it suffices to say that routines, especially those that are goal-directed, are performed based on predicted or expected outcomes, which can allow for adaptation to changes in context (Mendelsohn, 2019). Yet largely, the literature seems to overlook the micro habits formed before, during, and after routines and the associated physiological responses.

Micro habits refer to the smaller, often unconscious habits that are interwoven with or surround broader habits and routines (Shnayder-Adams & Sekhar, 2021). These micro-habits operate as subtle, supportive behaviours that reinforce or enable larger patterns of behaviour, often part of a larger habit cluster. For example, while the habit might be checking your phone notifications when you wake, micro-habits could include reaching for your phone, unlocking the screen, and expanding the notifications bar. By breaking habits down into their micro-habit components, it is easier to identify triggers, understand patterns, and make targeted changes. For instance, if you aim to reduce phone usage, you could start by interrupting just one micro-habit, such as placing your phone out of reach to delay the initial action of grabbing it.

Routines are argued to be planned and goal-oriented; however, habits through the mediating effects of micro-habits can reinforce or prevent the actualisation of routine-related goals. Using micro-habits to explain how humans respond automatically to environmental cues or stimuli allows us to detach from the cognitive burdens of routinised actions or behaviours. Also, adopting micro-habits has been argued as a necessary process in developing routines and adhering to them to achieve our intentions and goals (Shnayder-Adams & Sekhar, 2021). Once established, micro-habits can lead to incremental and significant changes in how habits are formed, characterised by repetitive goal-directed or routine actions; however, it can be uncomforting when this established pattern is disrupted. Consider this scenario:

Gym classes start at 9 am. Jenny arrives every day at 8.45 am to set up the weights and do a quick warm-up before the class starts. She always goes to the left-hand side and as close to the front of the class as possible. One day, she arrived and walked over to her usual spot, she realised someone else was setting up in her usual place. Moving to another available spot left her feeling displaced, inept, and uncomfortable.

This illustrates the complexity of routines and habits as interactive and interconnected subsystems within our human system. Reflecting on the above example, the complexities of psychological and physiological responses that occur as we move between intentional spaces present the challenge most of us face when forming and striving to continue new goal-directed behaviour.

## The interdependence of routines and habits

The literature often examines habits and routines as distinct constructs, typically treating them as separate entities (e.g., Cohn & Lynch, 2017; Ehn & Löfgren, 2020; Keller et al., 2021). Exceptions exist where studies explore the structures underlying these patterns (e.g., Reich & Williams, 2003) or attempt to empirically differentiate goal-directed behaviours from habitual responses (e.g., Gremel & Costa, 2013). However, such compartmentalised approaches risk overlooking the intricate dynamic interplay between routines and habits, which often coexist and mutually influence each other. For example, John’s daily routine of running laps at a specific time (time-bound) incorporated the habit of grabbing a towel from a fixed location (context-bound), illustrating their interconnectedness. Similarly, Jenny’s consistent gym attendance (routine) was complemented by her habit of standing in a specific spot (contextual). These examples emphasise that routines and habits are not mutually exclusive; instead, they reinforce each other in ways that are dynamic and evolving.

For instance, a routine of morning exercise might naturally incorporate the habit of laying out workout clothes the night before. Yet, the same routine might be undermined by a maladaptive habit, such as checking notifications during the exercise session, which diverts attention and disrupts flow. To elucidate these dynamics, Table 1 highlights key distinctions and intersections.

**Table 1:** Framing distinctions and interplay between routines and habits

Dimension	Routines	Habits	Interplay/Overlap
Trigger	Time-bound, deliberate (e.g., scheduled weekly meeting)	Context-bound (e.g., picking up a phone when it buzzes)	A time-bound routine can trigger context-bound habits (e.g., checking emails in a meeting)
Cognitive Effort	Requires conscious effort and planning (e.g., meal prepping)	Requires minimal cognitive input, automated (e.g., brushing teeth)	Routines can become automated, reducing cognitive effort over time
Goal Orientation	Explicit and goal-directed (e.g., exercising at 6 am to meet a fitness goal)	Often implicit and lacking explicit goals (e.g., nail-biting as a stress response)	Habits within routines can support or undermine explicit goals
Reflexivity	Reflexive and adaptable (e.g., adjusting a work schedule)	Rigid and non-reflexive (e.g., smoking when stressed)	Habitual actions may reinforce or disrupt flexible routines
Change Dynamics	Easier to modify with conscious effort (e.g., eating out instead of cooking)	Harder to modify due to automaticity (e.g., unlearning habitual behavior)	Habits embedded in routines can make routines harder to change

Routines, structured around temporal frameworks and often requiring conscious effort (Bernacer & Murillo, 2014), provide a deliberate scaffolding for habits to emerge. However, with repetition, some routines may become automatic, leading to reduced cognitive engagement, similar to habits. Habits, in contrast, are reflexive, context-driven actions triggered by environmental cues (Wood & Rünger, 2016). While temporal and contextual elements often overlap, since contexts inherently include temporal markers (Urcelay & Miller, 2014), the framework proposed here highlights the difference in cognitive engagement. While routines may contain elements of automaticity, particularly when performed frequently, they retain a degree of modifiable intentionality, allowing for adjustments when needed. Habits, on the other hand, operate on ingrained cue-response mechanisms, making them more resistant to deliberate change.

This viewpoint allows for an exploration of how repeated routines may transition into habits as their cognitive demands diminish over time. However, neuroscience complicates this perspective. For example, Mendelsohn (2019) argues that different neural pathways are activated during goal-directed routines (corticostriatal associative loop) versus habitual behaviours (corticostriatal sensorimotor loop). This distinction suggests a need for empirical clarity in behavioural models (Medhelsohn, 2019; de Wit et al., 2018). Nonetheless, this paper contends that while distinctions exist, the dual embedding of routines and habits within human systems challenges the notion of true separation. Over time, goal-directed behaviours can evolve into habits, sharing physiological responses and rendering clear distinctions less meaningful in practice.

## Human systems and sub-systems

Routines and habits are staple to humanity—environmentally, socially and biologically. In fact, we could say humans are passive recipients of a structured framework of fixed patterns, repetitions, and cycles, evident in the layout of our homes and the routines of our daily lives, such as changing streetlights, fashion trends, food choices, and work habits. These patterns, habits, and routines are shaped by our upbringing, social inclinations, and cultural influences, becoming more ingrained and entrenched with age, cultural norms and personal values. As a complex group of interacting systems, it is helpful to unpack what emerges when these routines and habits interact with other human systems.

Using a systems-thinking metaphor, understanding the complexity of routines and habits means appreciating that they are not isolated behaviours (Waldman, 2007); they are triggered, influenced, and reaffirmed by other rituals entwined or preceding them. Consider the habit of making a coffee at the same time every morning and what activities, feelings or thoughts may precede this coffee-making behaviour. For example, showering or bedmaking may be a routine before making a morning coffee, or the same feeling may be re-experienced each morning merely by thinking of a morning coffee.

In addition, other habitual behaviours may occur concurrently while performing the focal habit; these other habits, including micro habits, can be symbolic, without consciousness, yet embedded as part of our daily routine. For example, making the morning coffee on the same side of the bench, using a favourite mug, or adding coffee and milk before boiling water. This illustration highlights the multifaceted dimensions of what we may call a simple “habit”. Imagine if eliminating the morning coffee habit was now desired; all the other associated (secondary or symbolic) habits, thoughts and feelings could become part of the challenge to stop drinking coffee. Perhaps these secondary/symbolic habits are inherently part of the challenge to change what we perceive as simply a repetitive and isolated act.

Psychologically, we could say the sense of repetitive familiarity from habitual behaviours reaffirms our sense of self and sense of positioning or identity within our world. We quickly attach and identify ourselves with a routine or habit, for example, “I am a coffee drinker”, “I am a gym goer”, “I am a news watcher”, or “I am an early riser”. At times, the familiarity of patterned behaviour might not only be passively accepted but psychologically desired and craved, and at these times, constraint can be attributed to the loss of opportunities and serendipity. Nonetheless, if we accept that routines and habits provide a sense of power and control and are entwined with our sense of identity, then this interconnection means that deliberately shifting routines and habits can create psychological friction or dissonance (e.g., an identity tension) and discomfort, thus causing resistance to change.

The literature typically affirms the psychological underpinning of habits and routines (de Wit, et al., 2018). Yet, to fully unpack the complexity of routines and habits, we posit it is imperative to explore beyond the psychological realm, to unpack the congruency between physiology, psychology, and environments, and appreciate how complex these subsystems are as they interact and interconnect. Disentangling and understanding this complexity and interaction should be arguably the focus of future research on habits and routines.

The context or environment (e.g., entering the kitchen) energises or stimulates physiological responses or endorphin releases (e.g., a feeling of pleasure), thus triggering habitual behaviour (e.g. eating); this reaffirms a psychological response (e.g. comfort). Even when the physiological responses are less desired, the body can become acclimatised to the same sensation or physiological feedback loop. These physiological responses could be likened to a manifestation of addiction, where physiologically, and often beyond our state of consciousness, we crave the same physiological effect or reward from said repetitive patterns. The limited ability to adjust predetermined physiological responses could explain why adjusting behaviours creates psychological tensions and conflicts with the body’s attempt to maintain equilibrium. As a result, while we can cognitively decide to shift behaviours and routines through mindset adjustments and denial, this may be untenable long-term if it does not entice the same or stronger physiological responses, causing a sensation of loss or pleasure. As a result, we often fail to adopt new routines and habits.

Thus, so far, we have argued that the intricacy of routines and habits is more palpable when we consider their interplay with physiological, psychological, and environmental factors, which can include economic, and social contexts. For example, consider social dimensions, whereby behaviours and repetitive activities are in sync and entwined with each other’s movements, such as going to the gym with a friend or visiting the same café regularly with a spouse. These habits and routines are reinforced by the positive physiological feedback or endorphins we get with social interactions. While shifting or disrupting routines and habits can be more challenging when interlinked socially, this social connection can also be leveraged to reinforce positive patterned behaviours.

Yet fundamentally, the important question lies with how habits and routines can be broken, shifted, or adjusted for change and new opportunities when they are grounded in our physiological and psychological makeup. As a result, changing or adjusting our environment to stimulate strong physiological emotive responses could be a catalyst for successful habitual changes. The relevance of changing or reducing our routinised behaviours is further discussed in the next section.

## Paradox of habits and routines

The above discussion suggests that routines and habits create tensions on a continuum. On the one hand, scholars have argued that routinised-habitual behaviours are advantageous when they lead to efficiency, goal achievement, healthier lifestyles, and reduced stress (Reich & Williams, 2003). Following this school of thought, we could argue that routines play a role in maintaining repetitive behaviour (good or bad), reducing uncertainty-related stress, requiring less cognitive energy, reinforcing boundaries, and creating productivity through systemisation. Moreover, we can all recognise times when a patterned prearranged lifestyle can positively reduce distractive opportunistic behaviours and procrastinate activities that may deter us from achieving goals and aspirations.

On the other end of the continuum, there is an apparent paradox in that repetitive behavioural lifestyles, while often associated with efficiency, stability, and even progress, can simultaneously inhibit change, even when such change is beneficial or necessary for innovation and creativity. This contradiction arises because routines, which are typically seen as productive and goal-oriented, can paradoxically suppress curiosity and adaptability, making it easy to stagnate despite the need for growth. Even routines that support goal achievement can form a psychological safety net, as knowing what is happening and when requires less cognitive processing. While this predictability fosters comfort and efficiency, it can also lead to an overreliance on familiarity, reducing openness to novel ideas and experiences. In this way, the very structure that enables stability can, at the same time, act as a constraint, preventing the flexibility and openness required for meaningful change.

Further, it could be said that routine-based lifestyles promote reactive behaviours, whereas non-routine-based lifestyles promote proactive behaviours. Based on this perspective, routines and habits can be at odds with change and new environments, so much so that in times of dramatic change, people rapidly shift to establishing or reinforcing routines and habits to rediscover a sense of solace, grounding, and well-being. Since humans crave the effect of a comfort-seeking loop and are subjected to a franchised systemised lifestyle, we need to understand how the disruption of change and liminal thinking (Jeremiah et al., 2020) can be embraced for innovation, creativity, and progression.

A renowned behavioural scientist, Sutherland (2019), asserts that creativity and innovative ideas rarely come from the sequential, rational, linear process of a routine mindset. He further argues that creative breakthroughs seldom occur with intentional, sequential deployment of logical induction (Sutherland, 2019). In line with this thinking, we argue that novel ideas emerge from unexpected observations, serendipitous “eureka” moments, or as a beneficial consequence of unintentional outcomes. Yet cultivating a more oriental mindset requires sporadic changes in behaviour, allowing ambiguity over certitude in one’s lifestyle and managing the psychological tensions created by routines and habits.

While routinised behaviours often enhance efficiency and reduce cognitive load, they can simultaneously inhibit creativity and innovation. Sutherland (2019) suggests that creativity thrives in conditions of ambiguity and disruption, which contrast the predictable and orderly nature of routines. This aligns with the broader argument above that routines create psychological safety nets, reducing stress but potentially stifling serendipity and exploration.

However, routines may also enable creativity by freeing cognitive resources. For instance, habitualised elements of a routine (e.g., automated morning tasks) allow individuals to focus their mental energy on complex, novel problems. Thus, routines may paradoxically act as both a constraint and a catalyst for creativity, depending on the broader context and the degree of rigidity within the routine. Addressing this tension, future research could explore conditions under which routines foster or inhibit innovation and identify strategies to balance these opposing forces.

Key to navigating this way of thinking is cognitively reframing the role of patterned behaviour as the basis, structure, safety net and foundation from which we can let disorder unfold as interdependent but critical substrates of progression. Metaphorically, we could liken routines and habits to a launch pad; it is stable and secure and provides the certitude of solid grounding for high dynamism (rocket) to safely explore, change, risk, disrupt, and pioneer ambiguous opportunities. It is important to consider patterned behaviour as a foundation of certainty from which one can explore, especially in light of the stress caused by ambiguity. Stress can lead people to rely more on habits (Schwabe & Wolf, 2013), so by maintaining a level of certainty in our behavioural patterns, we can reduce stress and make it easier to break free from habits.

Fundamentally, routines and habits were more central and necessary in the industrial age; whereby monotonous factory line work superseded innovation and intrapreneurship. Yet today, it is likely that dominant routines can be counterintuitive in a technological-driven era that thrives on innovation, disruption, and rapid progression. Now that we encounter new challenges, new ways of thinking, new technologies, and a progressive digital error, it is essential to redefine and shift the way we perceive and position habits and routines as a supportive but non-restrictive, enfranchising platform.

Since we know there are ways to measure routines, it is possible to identify, with some surety, those who are more likely to live an innovative lifestyle. Research-led awareness affords the opportunity to adjust spaces that foster creativity, shake up routines, and challenge the established notion of automatic repetitive patterns.

## Routines and habits and our states of self

Understanding how routines and habits shape human behavior requires recognising their deep connection to self-identity. Repetitive behaviors do not only structure daily actions, but they also become embedded in how we define ourselves, influencing decision-making, adaptability, and the resistance to change. The States of Self framework can explain why some individuals find it easier to shift habits and routines, while others remain stuck in behavioral inertia. Based on this premise, we posit that there are three states of mind concerning action: the Self of Others (how others perceive us), the Self-Conscious Self (how we perceive/understand ourselves), and the Potential/Aspirational Self (our dream self).

The Self-Conscious Self seems to reflect the difference between the ‘Self Conscious Self ‘and the ‘Potential/Aspirational Self’. The “Potential/Aspirational Self’ is the perceived self of tomorrow; it is a futuristic self and may not be practical or realistic. We suggest that it is in these times that decisions are often made (for example, we may aspire to go to the gym or start a diet tomorrow); however, when tomorrow comes, we seem to be still stuck with the ‘Self Conscious Self’, or the non-potential, non-aspirational self. It is here we are reminded of the great chasm that exists between these two—a phenomenon known by behavioural scientists as the ‘Intention-Action Gap’ (Godin & Conner, 2008; Rabbi & Dey, 2013; Rhodes & Bruijn, 2013). This tension was described by Kentaro Fujita, a psychologist at Ohio State University, as: “our prototypical model of self-control is an angel on one side and devil on the other, and they battle it out”. Therefore, if the ‘Potential/Aspirational Self’ is all about decisions and actions (change), then the ‘Self Conscious Self’ is all about existing through a repetitive method—manifested in habits and routines. This is what unfolds when we remove aspirations and potential.

This perspective helps explain why behavioral change is often difficult—habits are not just actions but self-reinforcing identities. The challenge is not merely breaking a routine but redefining how one sees oneself within that shift. This insight becomes particularly relevant when considering strategies for habit transformation.

## Strategies for advancing adaptability and innovation

As we have explored, routines and habits exist as dynamic constructs that can both enable and inhibit adaptability, shaping behavior across innate, natural, and self-constructed cycles. While these patterns provide stability, they can also reinforce rigidity, making it difficult to embrace change in rapidly evolving environments. The challenge, then, is not just in understanding these cycles but in learning how to consciously reshape them. Next, we examine strategies for fostering adaptability and innovation, addressing how individuals and organizations can leverage the structure of habits while avoiding their constraints.

Turning to the literature, contemporary research underscores the pivotal role of physical and social environments in shaping cognitive processes, emotional resilience, and the disruption of maladaptive routines (Dul & Ceylan, 2011; Smith & Bond, 2022). One extensively documented strategy is biophilic design, which integrates natural elements into built spaces to reduce stress and stimulate adaptive behaviours (Kellert & Calabrese, 2015; Nieuwenhuis et al., 2014). Studies reveal that features like greenery, natural light, and organic textures not only lower stress markers such as cortisol levels but also enhance cognitive flexibility and emotional regulation, thereby supporting the recalibration of entrenched behaviours (Aries et al., 2015; Berman et al., 2008; Bratman et al., 2012). Exposure to natural light, for instance, has been shown to stabilize circadian rhythms, improve alertness, and counteract fatigue associated with static or overly artificial environments (Chan et al., 2023). These environmental interventions do more than modify surface-level behaviours; they act as catalysts for deeper psychological shifts, creating sensory conditions that disrupt habitual thought patterns and foster innovation in routines (Wilson, 1984).

The influence of physical spaces extends beyond their static features to encompass their capacity for dynamism. Even minor alterations in spatial layouts, such as rearranging furniture, repositioning decorative elements, or rotating artwork, have been shown to act as cognitive disruptors, challenging ingrained spatial assumptions and interrupting habitual behavioural routines (Kaplan, 1995; van den Berg & Custers, 2011). By introducing novelty into otherwise familiar contexts, such as standing instead of sitting at a desk, these modifications counteract the inertia of entrenched habits and encourage adaptive thinking. However, the efficacy of such interventions is contingent on their scale and pacing. Excessive or overly frequent environmental shifts risk overwhelming some, creating a sense of instability that undermines the intended benefits (Aries et al., 2015; Barrett et al., 2019). Instead, research advocates for incremental, thoughtfully timed changes that maintain a coherent sense of place while fostering an atmosphere of exploration and adaptability. This balance ensures that the environment supports both continuity and innovation, enabling individuals to recalibrate their routines without compromising psychological comfort (Rogan et al., 2022).

In tandem with physical adjustments, social dynamics play a pivotal role in disrupting routines and fostering adaptive behaviours. Accountability within social groups, such as partners encouraging each other to maintain daily exercise, exemplifies how collective engagement can reinforce positive behavioural shifts. Constructive competition further amplifies this effect by motivating individuals or groups to seek innovative strategies for achieving shared goals, thereby dislodging the inertia of long-standing practices (Deci & Ryan, 1985; Johnson & Johnson, 1989). Crucially, this process is most effective when participants are encouraged to experiment with novel approaches rather than rely on repetitive tactics, ensuring that competition becomes a driver of exploration and growth rather than mere replication (Tjosvold, 2007). Similarly, spontaneity within social interactions, facilitated by impromptu discussions in flexible spaces or chance encounters in communal areas, introduces unpredictability that challenges entrenched modes of thought (Janssen et al., 2004; Tjosvold, 2007). These unplanned interactions seemingly act as micro-disruptions, breaking rigid communication patterns and fostering cognitive flexibility essential for innovation (Meyers-Levy & Zhu, 2007).

Deliberately introducing novelty into daily routines serves as a powerful mechanism for disrupting maladaptive patterns by engaging the brain’s novelty circuits, particularly in the hippocampus and prefrontal cortex. These regions mediate dopamine release, which heightens motivation and fosters curiosity and adaptability (Krebs et al., 2009; Bunzeck & Düzel, 2006). Novel experiences, such as altering commuting routes or adopting new hobbies, disrupt repetitive behavioural loops, creating cognitive interruptions that counteract the automatic reinforcement of undesirable habits (Verplanken & Wood, 2006). Complementing these strategies, increasing friction for maladaptive behaviours while reducing it for desired ones amplifies their impact. For instance, making unhealthy snacks less accessible, employing app blockers to limit social media usage, or automating bright lights to coincide with a morning alarm subtly raises the effort required to sustain undesirable habits, steering behaviour toward more adaptive alternatives (Duckworth et al., 2016).

Mindfulness and meditation elicit profound physiological and neurological responses, making them powerful tools for habit transformation. Regular mindfulness practice reduces cortisol levels, enhances vagal tone, and increases prefrontal cortex activity, which governs self-regulation and emotional control (Brewer et al., 2011; Goyal et al., 2014). These processes trigger serotonin release, fostering a sense of calm and well-being that reinforces mindfulness as a habit. Complementary to mindfulness, structured breathwork practices such as diaphragmatic or alternate nostril breathing help regulate the autonomic nervous system by lowering sympathetic activity and increasing parasympathetic dominance, thereby reducing anxiety and promoting a sense of control (Brown & Gerbarg, 2013; Jerath et al., 2015).

Behavioural interventions that align actions with personal values provide additional pathways for disrupting maladaptive routines. Writing a commitment letter or reflecting on how a behaviour aligns, or conflicts with core values leverages the psychological drive for internal consistency, creating discomfort that motivates change (Festinger, 1957). Self-monitoring practices, such as tracking health-related behaviours, amplify this effect by increasing awareness and providing real-time feedback. Meta-analyses have demonstrated significant effect sizes (0.40–0.70) for self-monitoring in promoting habit change, driven by the combination of increased awareness and the positive reinforcement of progress tracking (Burke et al., 2011; Michie et al., 2011).

The above strategies provide valuable starting points for disrupting maladaptive patterns and fostering sustainable behavioural change. However, their effectiveness is context-dependent, often influenced by individual differences, environmental factors, and the interplay of intrinsic and extrinsic motivators. Future research could explore how these interventions can be tailored to diverse populations, including those facing unique cognitive, emotional, or environmental constraints.

## Researching habits and routines

### Propositions for future research

This conceptual discourse underscores the importance of viewing routines and habits as dynamic, interactive processes within the human system, analysed through the broader lens of systems thinking. This perspective invites a critical question: If one habit or routine is altered, how would the change cascade through the broader system? For example, would a new habit emerge to fill the time previously spent making and drinking coffee, and what implications would this substitution have for other behavioural patterns? Such questions underscore the need for future research to apply systems thinking to empirically investigate habits and routines, particularly within the digital and physical contexts where they coexist and overlap.

To illustrate this overlap, examining the consequences of banning social media usage (a digital context) and implementing open-plan office layouts (a physical context) on workplace productivity habits could illuminate how environmental and technological shifts influence behavioural systems. Also, AI tools like ChatGPT are increasingly altering workplace routines by automating repetitive tasks, undoubtedly shaping new habitual behaviours while displacing others. A research inquiry may ask, “How does AI and automation disrupt established patterns by taking over repetitive tasks, and how might this reshaping of roles cascade into other subsystems of behaviour”? Leveraging wearable technology to track movement patterns, along with digital activity logs, would provide a unique view into habit formation and disruption. Such cascading effects may reveal how changes in one subsystem reverberate across others, enabling interventions that optimise beneficial patterns while minimizing maladaptive ones.

Another promising avenue for research is the interplay between time-bound and context-bound behaviours. Understanding how temporal routines (e.g., daily commutes) intersect with contextual factors (e.g., cognitive demands or stress levels) could provide nuanced insights into the factors shaping overall well-being and productivity. For instance, examining how commuting routines influence cognitive performance and stress could reveal critical touchpoints for intervention. Furthermore, future research should investigate the extent to which habits are tied to classical conditioning, where unrelated environmental stimuli or cues unconsciously trigger specific behaviours. This would deepen our understanding of how limited cognitive control over certain actions may arise, as well as the conditions under which these automatic responses are most likely to occur (Akpan, 2020).

To fully capture the complexities of routines and habits, future studies must also explore the congruence between physiological, psychological, and environmental factors. These investigations should encompass the interconnectivity of habits and rituals, whether cognitive, affective, or behavioural, and their embedding within routinized or goal-directed behaviours. For instance, research could examine how environmental cues such as lighting, spatial design, or ambient noise influence physiological and psychological responses, stabilizing or disrupting habitual patterns.

A particularly valuable focus for future research lies in understanding how new or altered environments can disrupt maladaptive cyclic behaviours. We posit that creating environments that replicate the same physiological pleasure or arousal associated with entrenched routines (e.g., moving to a new city or fostering novel social interactions) is critical to breaking these patterns. For example, studies could investigate how the physiological effects of physical exercise, such as changes in heart rate or hormone levels, align with psychological states like motivation and stress, and how these alignments are influenced by environmental factors such as gym layouts or outdoor settings. Such findings could inform the design of interventions that not only disrupt maladaptive habits but also cultivate resilient, adaptive routines.

## Future research methodology

Measurement is the first step that leads to control and eventually to improvement. If you can’t measure something, you can’t understand it. If you can’t understand it, you can’t control it. If you can’t control it, you can’t improve it. (Harrington et al., 2016, p.363).

While researching patterned behaviour contributes to understanding the tension between routines and habits, social and behavioural investigators have adopted different research designs. For example, scholars have employed experiments (e.g., Quinn, 2010) or diary notes (Wood et al. 2002), self-reporting (e.g., Galla & Duckworth, 2015), and routines measured through tracking devices (e.g., Lima et al., 2016).

While self-reporting studies are the most popular method in this context (Wood & Rünger, 2016), they can have conflicting measures when cross-checked; recording participants’ actions through digital traces affords more accuracy. Indeed, this inconsistent data is arguably one of the most fundamental aspects underlying behavioural economics, which continues to portray how subconscious behaviours often vastly contradict self-perception (Wood & Rünger, 2016) and that humans behave irrationally and counterintuitive to benefits gained from rational behaviour (Ariely & Jones, 2008) such as those that might be gained through spontaneous exploratory behaviours.

Future research could build on this paper’s framework by adopting multimodal methodologies to investigate the nuanced interplay between routines and habits. By integrating physiological, psychological, and social dimensions, such approaches could empirically disentangle how routines and habits function as distinct yet interconnected constructs within human systems. For example, wearable devices might measure physiological states (e.g., heart rate variability) during routine and habitual behaviours, revealing differences in physical responses linked to deliberate versus automatic actions. Concurrently, the sensemaking approach could explore reflexive responses to habitual cues, such as snack consumption in workplace settings, and their contrast with goal-directed routines. Social network analysis could further illuminate how peer interactions and cultural norms reinforce or disrupt routines and habits within broader systems.

These multimodal approaches are particularly well-suited to investigating transitions—how routines evolve into habits or how micro-habits influence larger behavioural patterns over time. This perspective aligns with a systems-thinking lens, emphasizing the interconnected nature of physiological, psychological, and environmental influences on human behavior. For instance, longitudinal studies combining biometric data with artificially intelligent diaries could reveal the mechanisms underlying these transitions, offering critical insights into their adaptive potential. This perspective aligns with a systems-thinking approach, emphasising the interconnectedness of physiological, psychological, and environmental subsystems in shaping human behaviour. By leveraging these methods, future research could refine theoretical distinctions, resolve debates about the overlap between time-bound and context-bound behaviours, and inform interventions designed to optimise behavioural change.

## The relevance of understanding routines and habits

In this paper, we explored the role of unpacking the landscape of routines and habits in today’s current dynamic, digitally driven environment. The historically developed habits and routines (developed under the old non-digital paradigm) are now operating within a new (digital) paradigm which challenges current scholars to appreciate the duality of the physical and digital contexts in which habits and routines sync and overlap into each space.

Researching habits and routines can unlock an applied aspect of social science research, which can offer a practical advantage in addition to theoretical gains. For example, such an understanding of a patterned, rigid or constrained lifestyle can provide the space from which we can question whether our routines and habits are adaptive or maladaptive to our aspirations. As a result, understanding procrastination and other adverse cognitive, affective, and repetitive behaviours can contribute to advancement, success, and aspirational achievement, whether it is improved well-being or financial, professional, or academic advancement. It seems many people are not fully aware of how rigid their routines are; how much they work, learn, and socialise in the digital space; how little they expand outside of their rituals; or the habits they are unaware of doing and, in fact, may be far less efficient that they like to think (e.g. the amount of time spent on social media).

What is more, when individuals are aware of the full extent of their routines and habits, this may influence them to change their movements, increasing the potential for opportunities and a shift from the mundane to transformation. Awareness also provides the space to decide whether some habits and routines are beneficial, whereby the maintenance of stable patterns are related to multiple facets of health and well-being (Reich & Williams, 2003), or whether they are maladaptive, such as when some habits are undesirable (e.g. compulsive behaviour disorders) (Mendelsohn, 2019), or when habits can disrupt our aspirations and goals if they become too entrenched (Mendelsohn, 2019) or when an excessive amount of social media use leads to depression (Lin et al., 2016), or when mindsets, or affirmative repetitive thought patterns, are maladaptive when they negatively affect mood or cognitive reasoning (Watkins, 2008).

## Conclusion

In this paper, we constructed a new conceptual framework around routines and habits, arguing that applying a psychological lens alone in research overlooks their multifaceted nature. We conceptually packaged three overarching rotations that human existence is coupled to: innate, natural, and constructed cycles. We argue that a systems thinking framework is imperative to consider when researching cyclic behaviours, as it recognises the intricacies and coexistence of these fixed and dynamic patterns that influence the ability to shift routines and habits consciously.

We highlighted the paradox of routines and habits. While they can positively inhibit opportunistic and procrastinate behaviours as potentially distractive to aspirations, they can also neglect the importance of the mundane to transformation. Thus, we assert that our reconceptualisation of routines and habits is more applicable to the modern dynamic times of the digital age, which require flexibility from traditional routines and habits to allow for transformation, serendipity, and innovation.

## Data Availability

No datasets were generated or analysed during the current study.
